# Association of nutrition club membership with markers of health: a cross sectional study

**DOI:** 10.1186/s12889-017-4219-6

**Published:** 2017-04-11

**Authors:** Sai Krupa Das, Taylor A. Vail, Namibia Lebrón-Torres, Kara A. Livingston, Susan B. Roberts, Gail T. Rogers, Cheryl H. Gilhooly, Lorien E. Urban, Edward Saltzman, Nicola M. McKeown, Sara C. Folta

**Affiliations:** 1 Jean Mayer USDA Human Nutrition Research Center on Aging at Tufts University, 711 Washington Street, Boston, MA 02111 USA; 2 Gerald J., Dorothy R., Friedman School of Nutrition Science and Policy, Tufts University, Boston, MA USA

**Keywords:** Obesity, Nutrition assessment, Metabolic syndrome, Program evaluation, Health promotion

## Abstract

**Background:**

Nutrition clubs (NC) operate in community settings and provide members with nutrition education and meal replacements for weight management. NC are owned and operated by distributors of Herbalife products. There are over 6200 NC in the US, but there has been no independent assessment of the association of these NC with biomarkers of health.

**Methods:**

We conducted a cross-sectional pilot study to compare the health status of 100 NC members to 100 community-matched controls (CC) in the greater Boston area. Each CC was matched to a NC member for community of residence (zip code), age category, gender, BMI category, race/ethnicity, education level (category), and readiness to make health changes. Measures obtained included cardio-metabolic risk factors, body composition, markers of nutritional status, reported health status, dietary intake, physical activity, sleep and depression.

**Results:**

Participants were predominantly female (64%) and Hispanic (73%). NC members had significantly lower fasting insulin (*P* < 0.001) and lower HbA1c (*P* = 0.008), higher levels of 25 hydroxy-vitamin D (*P* = 0.001), and vitamin E:cholesterol ratio (*P* < 0.001), and lower prevalence of metabolic syndrome (*P* = 0.02) compared to CC. In addition, most of the NC members (99%) were satisfied with Herbalife NC membership for themselves and their families. A higher percentage of NC members (86%) compared to CC (32%) reported being in much better or somewhat better health compared to a year ago (*P* < 0.001); and they reported significantly better physical health (*P* = 0.03), and fewer sleep problems (*P* = 0.03).

**Conclusion:**

Herbalife NC membership was positively associated with perceived health and measured cardiometabolic benefits. However, causality cannot be inferred from these findings.

## Background

The obesity epidemic has persisted despite increased resources aimed at combating it [1, 2]. People with excess body weight are more likely to develop a range of co-morbidities, including diabetes and cardiovascular disease, resulting in high personal costs in terms of excess work-lost days, restricted activity, bed-days, and physician visits; as well as high societal costs, with obesity-related conditions representing nearly one-fifth of U.S. national health expenditures [3]. Even moderate weight loss is associated with improvements in health status including glycemic benefit and reductions in cardiovascular risk factors, namely,﻿﻿ blood pressure and blood lipid levels [4–7]. Recently, researchers have called for the systematic evaluation of existing community-based weight loss programs, including commercially available ones that reach larger numbers of people, to better understand the range of strategies for addressing the issue [1], particularly in populations at high risk for obesity and its related co-morbidities, often, the most difficult to reach with public health efforts.

In evaluating commercial weight-loss programs, it is important to determine their effect on cardiometabolic outcomes, to assist clinicians in determining which programs, if any, to recommend [8, 9] as well as helping to establish their potential public health impact. Programs vary in terms of meeting evidence-based recommendations, such as high-intensity counseling, prescription of a moderately reduced-calorie diet, increased physical activity, and behavior change strategies [5, 9], and therefore may vary in effectiveness at impacting both weight and cardiometabolic outcomes.

Herbalife is a global nutrition and weight management company http://www.herbalife.com */*. Herbalife products are sold exclusively by independent distributors, many of whom run Nutrition Clubs (NC). It is estimated that there are over 6200 NC in the US that provide members with prepared Herbalife products and facilitate activities, including exercise classes, nutrition coaching, weight loss education and social activities. NC aim to facilitate weight loss through the provision of calorie-controlled food products, some of which are designed to serve as meal replacements. Despite growing anecdotal evidence of weight-loss success in NC, their effectiveness has not been evaluated. Given the broad reach of NC, which extends to communities at high risk for obesity, it is important to understand their potential public health impact by examining behavioral and cardiometabolic outcomes. Since products are fortified, it is also important to understand their impact on nutritional status. Our objective was to conduct a pilot cross-sectional evaluation of the health status of Herbalife NC members as compared to community-matched controls (CC) in the greater Boston area.

## Methods

### Study participants

This study examined 100 Herbalife NC members in the greater Boston, Massachusetts, USA, area and 100 matched CC during the period of August 2014–June 2015. Each NC member was matched with a CC based on seven criteria: community of residence (zip code), self-reported interest in making healthful changes, age group (18-45 yrs. vs. > 45 yrs), race/ethnicity, sex, BMI category (normal weight vs. overweight and obese), and education (secondary or less vs. more than secondary education). Proportional sampling per club was used based on NC size and targeted enrollment numbers.

### Nutrition club and participant recruitment

Fourteen registered non-residential NC within the greater Boston area were invited to participate in this pilot study. Club operators were required to sign a memorandum of understanding permitting researchers to recruit a random sample of members and use club space for assessments. Eight NC expressed interest, and six signed the memorandum and an informed consent form (ICF) to participate in the study since they provided data about themselves and their clubs. For recruitment, club operators provided researchers with a list of members who were verified as having attended the NC at least once weekly for the past four months. From this list, researchers selected a random sample of 100 NC members for recruitment into this study. This random sample was weighted by size of each NC and accounted for a 20% refusal rate and ineligibility. CC were recruited from within the same zip code as the NC, and recruitment methods included distributing flyers, word of mouth, and online postings. Three hundred and twenty four potential CC were contacted and screened to obtain the 100 matched CC who were enrolled in the study. Recruitment for both the NC and CC groups were conducted simultaneously however, such that each CC was always recruited as soon as the NC for whom they were being matched was recruited.

Eligibility criteria included the following: BMI ≥ 18.5 kg/m^2^, non-pregnant, an interest in improving overall health, ≥18 years old, no speech or hearing impediment that would severely impact ability to participate, and literacy (in Spanish or English) to read and understand study documents. In addition, CC were required to have never participated in a NC or have consumed Herbalife products prior to and during their enrollment in the study and readiness to make changes to their health which was ascertained via a screening questionnaire based on stages of change. Potential CC were eligible if responses placed them in the contemplation or preparation stages [10].

### Study overview

Participants were required to attend up to three study visits within a two-week period during which all outcomes were obtained and data were recorded electronically by trained, bilingual (English and Spanish) researchers from the Jean Mayer USDA Human Nutrition Research Center on Aging (HNRCA) at Tufts University. Study materials and ICFs were translated into Spanish and back translated for assurance of fidelity. This study was reviewed and approved by the Tufts University Health Sciences Institutional Review Board, and all participants signed an ICF. This study was sponsored by Herbalife International of America; however, study investigators were ensured complete independence both during the data collection and analysis process per contractual agreement between Tufts University and the sponsor, including for publication of the results regardless of the nature of the findings. Further, de-identified data was analyzed by statistical experts not involved in data collection or with the funding source.

### Outcome measures

A demographic questionnaire was developed to capture age, gender, race and ethnicity, income, education and employment status and other health-related variables. NC participants were asked to indicate their primary reason for joining a club and their level of satisfaction with it (from “very satisfied” to “very dissatisfied”). Height was measured using a Seca214 mobile stadiometer. Weight and percent body fat were assessed using a Tanita TBF300A mobile body composition analyzer. Body Mass Index (BMI) was calculated as weight (kg) divided by height (m^2^). Waist and hip circumference were measured using a Seca201 flexible tape measure. Participants were requested to remove any extraneous clothing around the waist and hip, such as a jacket, that may hinder a proper measurement and to facilitate access to the waist and hip. Natural waist circumference was measured as the narrowest part of the torso (between the lower margin of the lowest rib and the top of the iliac crest), and hip circumference was measured as the maximum circumference of the buttocks. The tape was positioned in a horizontal plane at the identified landmark. The waist measurement was obtained at the end of normal expiration, and the hip measurement was obtained with the participant standing relaxed, with feet positioned together and weight evenly distributed across the feet. Readings for waist and hip were obtained to the nearest 0.1 cm and the average of the two measurements agreeing within ±1.0 cm was used as the final value.

Blood pressure was measured using the Omron HEM-705CP Digital Blood Pressure Monitor, according to the American Heart Association’s Guidelines [11].

Health status outcomes were captured using the short form (SF) 36 General Health Questionnaire [12]. Norm-based summary scores were calculated for the following eight health domains: general health, bodily pain, mental health, physical functioning, role limitations due to emotional problems, role limitations due to physical problems, social functioning, and vitality. Two component measures, physical health and mental health, were calculated to more broadly summarize the 8 domains.

Diet intake was assessed by the 2005 Block food frequency questionnaire (FFQ) [13, 14] and Alternative Healthy Eating Index (AHEI) used to estimate diet quality [15, 16]. AHEI is an 11 component score measuring diet quality, based on current scientific knowledge, and scores can range from 0 (worst/least healthy) to 120 (best/most healthy). Calculation of the AHEI was restricted to participants reporting energy intake 500 ≤ kcal ≤6000 per day. Dietary supplement use was defined as reported use of at least 1 to 3 times per week of the following: regular once-a-day, centrum etc., stress-tabs or B-complex vitamins, vitamin E, or vitamin D.

Physical activity was captured by self-report using the International Physical Activity Questionnaire (IPAQ) short form questionnaire [17], and objectively measured using the Yamax CW-701 Digiwalker pedometer which participants were instructed to wear on their waistband for seven consecutive days, removing them only when sleeping or showering.

Sleep as captured by the Medical Outcomes Study (MOS) 6-Item Sleep Scale Questionnaire [18] and depression using the Center for Epidemiologic Studies Depression Scale (CESD) [19] were assessed as potential covariates.

Fasting blood samples (>8 h) were stored on ice and processed within 3 hours. Samples were analyzed for glycated hemoglobin (HbA1C), insulin and glucose, plasma triglycerides, cholesterol (total, HDL and LDL), C-reactive protein (CRP), serum α-tocopherol, and plasma vitamin D.

We used three additional criteria to define metabolic health outcomes: (a) high cholesterol, defined as taking lipid-lowering medication or fasting LDL cholesterol >160 mg/dL or fasting total cholesterol >240 mg/dL; (b) elevated inflammation, as C-reactive protein > 3 mg/L; (c) metabolic syndrome per ATP 3 guidelines of having 3 or more of the following [20]: waist circumference > 102 cm for men and >88 cm for women; fasting plasma triglycerides ≥150 mg/dL or taking cholesterol-lowering medication; fasting HDL cholesterol <40 mg/dL for men or <50 mg/dL for women, or taking cholesterol-lowering medication; systolic blood pressure ≥ 130 mmHg and/or diastolic blood pressure ≥ 85 mmHg; or taking hypertension medication; or fasting plasma glucose ≥100 mg/dL or taking diabetes medication.

In addition, elevated HbA1c (%) was categorized as reflecting pre-diabetes, defined as a HbA1c >5.7 and ≤ 6.4% and diabetes defined as a HbA1c > 6.4%.

### Statistical analyses

This pilot study was designed to inform future, larger, prospective studies on the health status of community-based NC members, and no a priori power calculations were performed. All variables were assessed for normality prior to analysis. Comparisons of subject characteristics and self-reported health and measured indicators of nutritional status and metabolic health between NC and CC were performed using a Wilcoxon signed rank test for non-parametric outcomes, McNemar’s test for dichotomous outcomes, and paired t-test for normally distributed outcomes. If data were missing for any variable for either the NC or CC, data for the corresponding matched pair were excluded. Corresponding means ± SD, medians (range), and percentages are presented in Tables 1-2. Secondary analyses using paired logistic regression were performed to examine the association between individual factors (i.e. depression, physical activity, sleep, AHEI and supplement use) and metabolic syndrome (MS) in the presence of club status. Each factor was tested as a confounder and as an independent predictor in the model. All secondary models were adjusted for site, smoking status (y/n), and alcohol as a percent of energy.

**Table 1:** Participant characteristics of club members vs. controls

*N* = 100 club members, *N* = 100 controls (unless otherwise specified)	Club Members	Controls	*P*-value^a^
Site			
1	30 (30%)	30 (30%)	0.99^b^
2	27 (27%)	27 (27%)	0.99^b^
3	12 (12%)	12 (12%)	0.99^b^
4	13 (13%)	13 (13%)	0.99^b^
5	10 (10%)	10 (10%)	0.99^b^
6	8 (8%)	8 (8%)	0.99^b^
Age (yrs)	41.1 ± 10.9	39.7 ± 12.9	0.12
Gender (% male)	36 (36%)	36 (36%)	0.99^b^
Race/Ethnicity			
White	27 (27%)	27 (27%)	0.99^b^
Hispanic/Latino/Spanish	73 (73%)	73 (73%)	0.99^b^
Annual household income			
Less than $14,999	29 (29%)	34 (34%)	0.46
$15,000–$29,999	21 (21%)	20 (20%)	0.86
$30,000–$49,999	16 (16%)	20 (20%)	0.60
$50,000–99,999	18 (18%)	18 (18%)	0.99
$100,000–$149,999	10 (10%)	4 (4%)	0.15
$150,000+	6 (6%)	4 (4%)	0.73
Current Smokers (%)	6 (6%)	13 (13%)	0.17
Supplement Use	26 (26%)	21 (21%)	0.58
% self-reporting diabetes	11 (11%)	12 (12%)	0.81
% using medication for diabetes	7 (7%)	10 (10%)	0.55
% self-reporting high cholesterol	27 (27%)	12 (12%)	***0.008***
% using medication for high cholesterol	12 (12%)	9 (9%)	0.61
% self-reporting hypertension	8 (8%)	15 (15%)	0.17
% using medication for hypertension	5 (5%)	13 (13%)	0.08
% self-reporting heart disease	3 (3%)	1 (1%)	0.62
% using medication for heart disease	3 (3%)	0 (0%)	n/a
Highest level of education completed			
Never attended school	3 (3%)	1 (1%)	0.62
Elementary school	31 (31%)	22 (22%)	0.14
High school	30 (30%)	41 (41%)	0.05
Professional certificate or college 1–3 yrs	13 (13%)	19 (19%)	0.18
Bachelor’s degree	17 (17%)	13 (13%)	0.45
Graduate or professional degree	6 (6%)	4 (4%)	0.75
Health Behaviors			
SF36 Health Survey Measures (norm-based)^c^			
General health	58.2 ± 7.8	52.7 ± 10.8	***<0.001***
Bodily pain	54.7 ± 8.7	52.1 ± 10.6	***0.04***
Mental health	53.5 ± 9.1	51.0 ± 11.1	0.09
Physical functioning	55.0 ± 5.4	54.9 ± 4.6	0.78
Role limitations due to emotional problems	52.5 ± 7.9	51.8 ± 8.6	0.52
Role limitations due to physical problems	54.5 ± 6.6	53.8 ± 6.5	0.43
Social functioning	52.7 ± 7.4	51.4 ± 9.4	0.25
Vitality	57.3 ± 7.6	53.8 ± 9.4	***0.006***
Mental health component score	52.9 ± 8.3	50.7 ± 10.7	0.11
Physical health component score	56.2 ± 6.4	54.3 ± 6.8	***0.03***
AHEI score, median ± IQR (range)^d^	67.7 ± 13.4 (42.8–83.6)	60.4 ± 11.9 (35.5–80.2)	***<0.001***
Physical Activity			
IPAQ walking MET minutes/week, median (range)^e^	594 (66–11,088)	792 (23.1–11,088)	0.49
Total steps (per day)^f^	6310.7 ± 3540.2	5393.8 ± 4045.6	0.12
IPAQ summary score, median (range)^g^	3180 (132–27,090)	4293 (99–32,725)	0.27
MOS sleep summary score, median (range)^h^	52.2 (23.1–63.8)	50.2 (25.0–63.8)	***0.03***
Depression: CESD total score, median (range)^i^	7.0 (0–42)	6.0 (0–48)	0.45
% Depressed^i^	19 (20.0%)	25 (26.3%)	0.40

N (%), mean ± SD, or median (range)

*IPAQ* International Physical Activity Questionniare, *MOS* Medical Outcomes Study, *CESD* Center for Epidemiologic Studies Depression Scale, *AHEI* Alternative Healthy Eating Index

^a^Paired t test for age, SF36 health survey measures, food intake behavior, and family/friend encouragement, discouragement, and participation questions; Wilcoxon signed rank test for CESD, IPAQ, MOS sleep summary scores, and AHEI score; McNemar’s test for others. Significant *p*-values are indicated by italicized numbers in bold

^b^variable was part of matching criteria for study

^c^higher scores indicate better health/ better outcome (ie. better general health, less bodily pain, better mental health, etc.)

^d^AHEI is an 11 component score measuring diet quality, based on current scientific knowledge, and scores can range from 0 (worst/least healthy) to 120 (best/most healthy); assessed by Block Food Frequency Questionniare (*N* = 94 pairs), restricting to participants reporting energy intake 500 ≤ kcal ≤6000

^e^MET = ‘metabolic equivalent task’ which expresses the intensity of a physical activity; walking MET = 3.3 x walking minutes x walking days; thus, an individual walking 30 min per day for 7 days per week would be assigned walking MET = 3.3 × 30 × 7 = 693 MET minutes/week; *N* = 87 pairs

^f^ *N* = 88 pairs

^g^summary score is sum of MET minutes/week for walking, moderate, and vigorous activity; IPAQ assigns walking 3.3 METs, moderate activity 4.0 METs, and vigorous activity 8.0 METs; *N* = 97 pairs

^h^ *N* = 99 pairs; MOS sleep index score based on 6 components and is an overall measure of the extent/severity of sleep problems; higher score indicates fewer sleep-related problems

^i^ *N* = 95 pairs; score of less than 16 indicates no clinically significant depression, and 16 is sub-threshold for clinical depression

**Table 2:** Body composition and cardiometabolic health

Mean ± SD	Club Members	Controls	*P*-value^a^
Weight (kg)	80.2 ± 17.7	77.3 ± 19.1	0.07
Height (cm)	162.8 ± 10.0	162.0 ± 9.3	0.44
BMI (kg/m^2^)	30.1 ± 5.1	29.3 ± 5.8	0.07
% body fat^b^	35.5 ± 8.7	32.8 ± 8.6	***<0.001***
Diastolic blood pressure (mmHg)	79.5 ± 9.9	77.1 ± 11.3	0.05
Systolic blood pressure (mmHg)	127.0 ± 16.8	128.0 ± 17.5	0.60
Waist to Hip Ratio	0.87 ± 0.09	0.89 ± 0.09	***0.04***
Insulin (uIU/ml), serum^c^	7.9 (6.8, 9.1)	11.5 (9.9, 13.4)	***<0.001***
HbA1c (%), whole blood^d^	5.6 ± 0.7	6.0 ± 1.2	***0.008***
% Prediabetic (5.7 < HbA1c ≤ 6.4)	21.0 (21.9%)	24.0 (25.0%)	0.73
% Diabetic (HbA1c ≥6.4)	9.0 (9.4%)	20.0 (20.8%)	***0.03***
N (%)	Club Members	Controls	*p*-value^e^
Metabolic Syndrome^f,g^	23 (24.2%)	36 (37.9%)	***0.02***
Large waist circumference^f,h^	55 (57.9%)	55 (57.9%)	0.99
High fasting triglycerides^f,i^	20 (21.0%)	29 (30.5%)	0.17
Low fasting HDL cholesterol^f, j^	28 (29.5%)	38 (40.0%)	0.16
Elevated blood pressure^f,k^	38 (40.0%)	45 (47.4%)	0.26
High fasting glucose^f,l^	22 (23.2%)	30 (31.6%)	0.20
High Cholesterol^m^	16 (16.7%)	11 (11.5%)	0.38
Elevated CRP^n^	26 (27.1%)	35 (36.5%)	0.17

^a^paired t-test used to determine significance; 100 club members, 100 controls. Significant *p*-values are indicated by italicized numbers in bold

^b^ *N* = 93 pairs

^c^ *N* = 94 pairs

^d^ *N* = 96 pairs, Elevated HbA1c, reflecting pre-diabetes, was defined as 5.7 < HbA1c ≤ 6.4%

^e^McNemar’s test used for metabolic syndrome and its components, high cholesterol, elevated CRP, and elevated HbA1c; paired t test used for total metabolic health score; Significant *p*-values are indicated by italicized numbers in bold

^f^ *N* = 95 pairs;

^g^Based on the ATP 3 guidelines of having 3 or more of the following;

^h^waist circumferences of >102 cm for men and >88 cm for women;

^i^fasting plasma triglycerides ≥150 mg/dL or taking cholesterol lowering medication;

^j^fasting HDL cholesterol <40 mg/dL for men or <50 mg/dL for women, or taking cholesterol lowering medication;

^k^systolic blood pressure ≥ 130 mmHg and/or diastolic blood pressure ≥ 85 mmHg, or taking hypertension medication;

^l^fasting plasma glucose ≥100 mg/dL or taking diabetes medication;

^m^N = 96 pairs, High cholesterol was defined as taking lipid-lowering medication or having fasting LDL cholesterol >160 mg/dL or fasting total cholesterol of >240 mg/dL;

^n^ *N* = 96 pairs, Elevated CRP was defined as >3 mg/L

Nutritional biomarkers were assessed using a linear mixed model with pair ID as a random effect to account for the paired nature of the data. Models were adjusted for site, smoking status (y/n), alcohol intake (as percent energy), and dietary supplement use. An additional adjustment for season was included in the Vitamin D 25 (OH) analysis. All nutritional markers were positively skewed; therefore, outcomes were logarithmically transformed, and the geometric means and 95% confidence intervals are presented (Table 3).

**Table 3:** Nutritional markers^a^

Geometric means and 95% CI	Club Members	Controls	*P*-value
α-tocopherol (μg/dL), serum	1275 (1202, 1354)	1157 (1090, 1229)	***0.009***
Serum Vitamin E: Total Cholesterol ratio	6.8 (6.5, 7.1)	6.0 (5.7, 6.3)	***<0.001***
Vitamin D 25(OH) (ng/dL), plasma^c^	27.3 (25.1, 29.8)	23.2 (21.2, 25.2)	***0.001***
N (%) deficient vitamin D using IOM (20 ng/mL) cutpoint^b^	13 (13.5%)	35 (36.5%)	***<0.001***

^a^adjusted for site, current smoker (y/n), alcohol intake, dietary supplement use (defined as reported use of at least 1 to 3 times per week of the following:regular once-a-day, centrum etc. ., stress-tabs or B-complex vitamins, vitamin E or vitamin D)

^b^not adjusted; 96 pairs; deficient defined as <20 ng/mL, *P*-value from McNemar’s test

^c^ vitamin D analysis additionally adjusted for season; *p*-values from Type III fixed effects ANOVA from a linear mixed model with pair ID as a random effect to take into account the paired nature of the data; *N* = 92 pairs

Significant *p*-values are indicated by italicized numbers in bold

All statistical analyses were performed using the Statistical Analysis System (SAS) version 9.3 (SAS Institute, Cary, NC, USA). Unless otherwise stated, statistical significance refers to *P* -values <0.05.

## Results

### Demographic and Participant Characteristics, NC vs. CC:

Sixty-four percent of NC members and CC were female and 73% were Hispanic. No significant differences in the matching criteria were observed (Table 1), or in income levels, smoking status, supplement use, self-reported diabetes or other self-reported cardiometabolic risk factors or disease between the NC members and CC, with the exception of self-reported high cholesterol (27% of NC vs.12% of CC, *P* = 0.008).

**Fig. 1:**
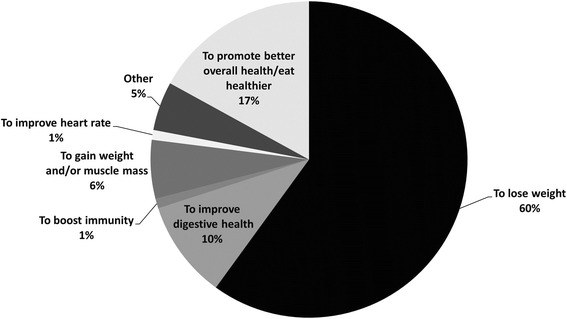
Primary reason for joining the club

**Fig. 2:**
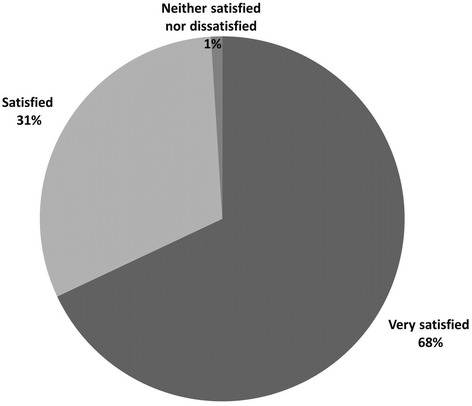
How satisfied are you with the nutrition club you have been attending

Wanting to lose weight was the primary reason for joining the NC for most members (60%), followed by ‘to promote better eating and overall health’ (17%) (Fig. 1). Ninety-nine percent of members reported being satisfied with their NC (“very satisfied” or “satisfied” combined). The remaining 1% reported being neither satisfied nor dissatisfied (Fig. 2).

### Self-Reported Health Status

**Fig. 3:**
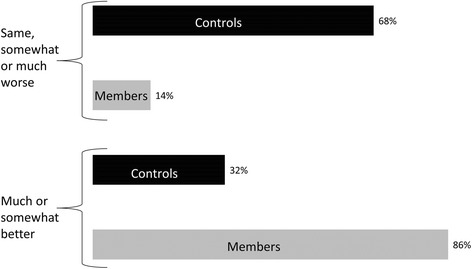
Self-reported health compared to one year ago

The majority of NC members and CC reported being in good overall health, however, NC members more frequently reported being in much better or somewhat better health compared to a year ago when compared to CC (86% NC vs. 32% CC, *P* < 0.001) (Fig. 3). NC members had significantly better scores for the general health (*P* < 0.001), bodily pain (*P* = 0.04), and vitality (*P* = 0.006) domains, but not for the physical functioning, physical, emotional, or social role functioning, or mental health domains. NC members had a significantly better physical health component score than CC (*P* = 0.03); however, no differences were observed for the mental health component score (*P* = 0.11).

### Diet and Physical Activity

Diet quality (AHEI score) was significantly higher in NC members compared to CC (67.7 versus 60.4, *P* < 0.001). NC members averaged 6310 ± 3540 steps per day, and CC averaged 5393 ± 4045 steps per day (*P* = 0.12) (Table 1). NC members reported fewer MET-minutes per week of both overall activity (3180 vs. 4233) and walking (693 vs. 792) compared to CC, however these differences were also non-significant (*P* = 0.27 and *P* = 0.49 respectively).

### Sleep and Depression

Based on an overall sleep summary score, NC members reported fewer sleep-related problems than CC (*P* = 0.03) (Table 1). No significant difference was observed in prevalence of depression (*P* = 0.40).

### Body Composition and Cardiometabolic Health

NC members had significantly higher percent body fat (*P* < 0.001) and marginally significant higher diastolic blood pressure (*P* = 0.05), but lower waist-to-hip ratio (0.87 vs 0.89, *P* = 0.04) than CC (Table 2). Due to the matching criteria, no significant differences were observed for weight or BMI. Fasting levels of HbA1c (%) was significantly lower in the NC compared to CC (*P* = 0.008). No significant difference in the prevalence of pre-diabetes was observed between NC members and CC (*P* = 0.73), but a significantly higher percentage of CC (20%) compared to NC members (9%) were diabetic (*P* = 0.03).

The prevalence of MS was lower in NC members than in CC (24% vs 38%, *P* = 0.02). No significant difference was observed between NC members and CC for the risk of elevated cholesterol or CRP. In secondary analyses, the odds of MS were 65% lower in NC members than in CC after additional adjustment for club site, smoking status, and alcohol intake (*P* = 0.02, data not shown). Depression, PA, sleep, AHEI, and supplement use were not independent predictors of MS. However, sleep and depression did slightly attenuate the association between MS and club membership (CESD OR 0.4, (0.14, 1.16), *P* = 0.09; Sleep OR: 0.41 (0.17, 1.03), *P* = 0.06).

### Nutritional Biomarkers

NC members had significantly higher levels of α-tocopherol (1275 vs 1157 μg/dL, *P* = 0.009), vitamin E:total cholesterol ratio (6.8 vs 6.0 *P* < 0.001), and plasma vitamin D (27.3 vs 23.3 ng/dL, *P* = 0.001) compared to CC (Table 3), in models adjusted for site, current smoker (y/n), alcohol intake, dietary supplement use, as well as season for Vitamin D. Using the clinical cutpoint of vitamin D deficiency defined as <20 ng/mL, a significantly higher proportion of CC were vitamin D deficient compared to NC members (37% versus 14%, *P* < 0.001, unadjusted).

Additional adjustment for AHEI in these models did not attenuate the association with Vitamin E: Total Cholesterol (6.7 NC vs. 6.1 CC, *P* = 0.002) or Vitamin D (27.1 NC vs. 23.5 CC, *P* = 0.007) but did for α-tocopherol (1253 NC vs. 1185 CC, *P* = 0.14).

## Discussion

This is the first study to examine the nutritional and health status of individuals who attend Herbalife NC in comparison to members in the community with broadly similar characteristics. Our independent assessment of the impact of these NC indicate that NC members have better perceived health and sleep, and higher levels of clinically measured nutritional biomarkers and better overall cardiometabolic health compared to community matched controls.

A high percentage (99%) of NC members reported being satisfied with their club membership and, similarly, being in better or somewhat better health compared to a year ago. This finding is consistent with the higher general health scores, better overall physical health, greater vitality, and less bodily pains combined with the trend for a higher mental health score and functional capacity in the NC members vs the CC. This finding is consistent with other studies involving group-based nutritional and behavioral interventions offered in community settings, in which improvements in these SF36 domains were observed [21, 22].

In terms of health behaviors, physical activity did not differ significantly between club members and controls. Both groups fell below the average 6500 steps per day reported by US adults [23] and well under the recommendation of 10,000 step per day required to meet physical activity guidelines [24]. While three of the six clubs studied offered opportunities for physical activity such as group exercise and Zumba classes, the main focus within the clubs studied was on product consumption and nutrition education. Club members may potentially benefit from an increased emphasis on physical activity.

NC members had slightly higher diastolic blood pressure than CC (although average values were within the clinically normal range), and had a higher percent body fat despite a lower waist to hip ratio and the non-significant difference in body weight. An important finding was the lower prevalence of MS in the NC members compared to the CC. However, no other measures of cardiometabolic health were significantly different between the NC members and CC. While cross-sectional, these results are reasonably consistent with findings from a recent systematic review examining the effects of commercial weight loss programs which showed limited effects on blood pressure and lipids [9].

With regards to nutritional status, both AHEI score and biomarkers of nutritional status were more favorable among NC participants, who had significantly higher levels of α-tocopherol and 25 hydroxy-vitamin D. Club members routinely consume several Herbalife products which contain appreciable amounts of α-tocopherol, a form which is preferentially incorporated into the plasma [25]. Vitamin E status itself was calculated as the ratio between vitamin E and total cholesterol (αT:TC) [26], and club members had a significantly higher ratio than controls. This may be due, in part, to the better diet quality reported by club participants.

Using the National Academies of Science, Engineering & Medicine cutpoint of vitamin D deficiency defined as <20 ng/mL [27], a significantly higher proportion of CC were vitamin D deficient compared to NC (37% versus 14%). Using the same cutpoint, NHANES 2005–2006 data indicate an overall deficiency prevalence of 41.6% among US adults and 69.2% among Hispanics [28]. While causality cannot be established in this cross-sectional study, given that the Herbalife products are fortified with vitamin D, these results suggest a benefit of product consumption on serum vitamin D levels.

This study had several strengths, including the capture of effects in a community setting using robust methodology. NC members were successfully matched with CC based on seven criteria to minimize the effect of potential confounders, in particular residual confounding. Although NC were self-selected, participants within clubs were chosen using random sampling to rule out provision by club operators of the most successful members. The reported health and behavioral data were substantiated by objective measures of fasted body composition, blood measures of nutritional status, and cardiometabolic health.

This study was designed as a pilot to inform future research, and there are some limitations to the design. The NC studied were based on a convenience sample of clubs within the greater Boston area, and the results of our study cannot be generalized to Herbalife NC nationally. Further, because there were no a priori power calculations, the study was not powered to perform multiple group comparisons. However, it must be noted that the highlighted associations in the paper are highly significant and likely to remain so even if corrected for multiple comparisons.

We used education as a proxy for income as a matching criterion and although we found no significant difference in income levels between NC and CC, we recognize that income is an important predictor of health status and should be considered for future matching. The cross-sectional design provides data on the health and wellness among participants compared to community-matched controls; however, no causal inference may be drawn.

## Conclusions

This study provides the first direct comparison of Herbalife NC members with CC and results from this cross-sectional study suggest that individuals participating in Herbalife NC have better perceived health and overall cardiometabolic health compared to CC. These pilot data suggest that the NC may have benefits for participants and justify further longitudinal studies to examine the effect of Herbalife NC membership on health and quality of life.

## Abbreviations

AHEI: Alternative healthy eating index
BMI: Body mass index
CC: Community-matched controls
CESD: Center for epidemiologic studies depression scale
CRP: C-reactive protein
FFQ: Food frequency questionnaire [13, 14]
HNRCA: Human nutrition research center on aging
ICF: Informed consent form
IPAQ: International physical activity questionnaire
MOS: Medical outcomes study
NC: Nutrition clubs
USDA: United States Department of Agriculture
